# 3-(2,6-Dimethyl­anilino)imidazo[1,2-*a*]pyridin-1-ium perchlorate

**DOI:** 10.1107/S1600536811014735

**Published:** 2011-04-29

**Authors:** Gary S. Nichol, Anuj Sharma, Hong-Yu Li

**Affiliations:** a Department of Chemistry and Biochemistry, 1306 East University Boulevard, University of Arizona, Tucson, AZ 85721, USA; b Southwest Center for Drug Discovery and Development, College of Pharmacy, BIO5 Institute, University of Arizona, Tucson, AZ 85721, USA

## Abstract

The structure of the organic cation in the title compound, C_15_H_16_N_3_^+^·ClO_4_^−^, contains two essentially planar rings. Mean planes fitted through all non-H atoms of each ring system have an r.m.s. deviation of 0.019 Å for the imidazole-based ring and 0.016 Å for the 2,6-dimethyl­phenyl ring. The angle between the two planes is 86.76 (2)°. In the crystal structure, N—H⋯O inter­actions form a one-dimensional chain, which propagates in the *b*-axis direction. C—H⋯O inter­actions are also found in the crystal packing.

## Related literature

For background information on the Groebke–Blackburn synthesis, see: Bienaymé & Bouzid (1998 ▶); Blackburn *et al.* (1998 ▶); Groebke *et al.* (1998 ▶). For details of the chemical synthesis, see: Nichol *et al.* (2011 ▶); Sharma & Li (2011 ▶). For information on graph-set notation to describe hydrogen-bonding motifs, see: Bernstein *et al.* (1995 ▶).
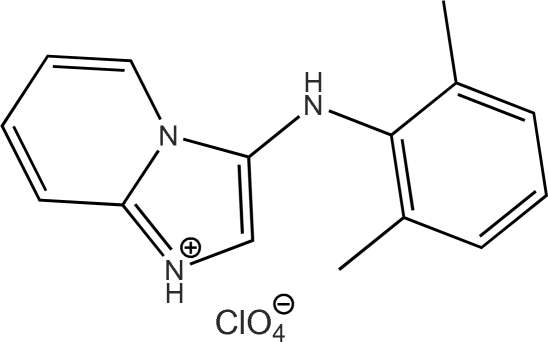


## Experimental

### 

#### Crystal data

C_15_H_16_N_3_^+^·ClO_4_^−^
*M* *_r_* = 337.76
Triclinic, 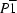

*a* = 8.6347 (3) Å
*b* = 8.7663 (3) Å
*c* = 11.5155 (4) Å
α = 70.668 (2)°
β = 73.131 (2)°
γ = 72.679 (2)°
*V* = 767.24 (5) Å^3^
*Z* = 2
Mo *K*α radiation
μ = 0.27 mm^−1^
*T* = 100 K
0.26 × 0.16 × 0.16 mm

#### Data collection

Bruker Kappa APEXII DUO CCD diffractometer
Absorption correction: multi-scan (*SADABS*; Sheldrick, 1996 ▶) *T*_min_ = 0.932, *T*_max_ = 0.957
27864 measured reflections
8640 independent reflections
6871 reflections with *I* > 2σ(*I*)
*R*_int_ = 0.031

#### Refinement

*R*[*F* ^2^ > 2σ(*F* ^2^)] = 0.038
*wR*(*F* ^2^) = 0.111
*S* = 1.05
8640 reflections
272 parameters
All H-atom parameters refined
Δρ_max_ = 0.61 e Å^−3^
Δρ_min_ = −0.51 e Å^−3^

### 

Data collection: *APEX2* (Bruker, 2007 ▶); cell refinement: *SAINT* (Bruker, 2007 ▶); data reduction: *SAINT*; program(s) used to solve structure: *SHELXTL* (Sheldrick, 2008 ▶); program(s) used to refine structure: *SHELXTL*; molecular graphics: *ORTEP-3 for Windows* (Farrugia, 1997 ▶) and *Mercury* (Macrae *et al.*, 2008 ▶); software used to prepare material for publication: *SHELXTL* and *publCIF* (Westrip, 2010 ▶).

## Supplementary Material

Crystal structure: contains datablocks I, global. DOI: 10.1107/S1600536811014735/kj2175sup1.cif

Structure factors: contains datablocks I. DOI: 10.1107/S1600536811014735/kj2175Isup2.hkl

Supplementary material file. DOI: 10.1107/S1600536811014735/kj2175Isup3.cdx

Additional supplementary materials:  crystallographic information; 3D view; checkCIF report

## Figures and Tables

**Table 1:** Hydrogen-bond geometry (Å, °)

*D*—H⋯*A*	*D*—H	H⋯*A*	*D*⋯*A*	*D*—H⋯*A*
N2—H2N⋯O1^i^	0.869 (16)	1.955 (17)	2.8169 (10)	170.8 (15)
N3—H3N⋯O3	0.832 (16)	2.216 (15)	2.8899 (10)	138.3 (14)
C2—H2⋯O2^ii^	0.911 (14)	2.547 (14)	3.3826 (11)	152.8 (12)
C3—H3⋯O4^ii^	0.971 (15)	2.559 (15)	3.2893 (12)	132.0 (11)

Symmetry codes: (i) 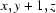; (ii) 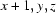.

**Fig. 1.**
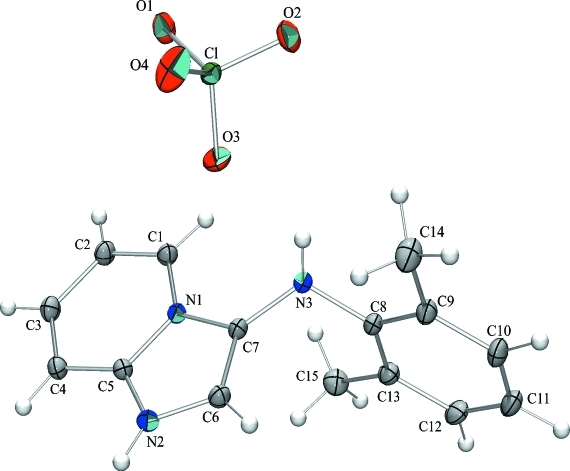
The asymmetric unit of the title compound with displacement ellipsoids at the 50% probability level.

**Fig. 2.**
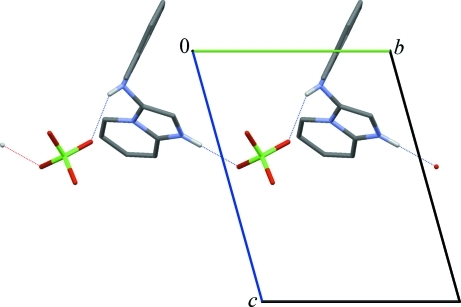
N—H···O interactions (dotted blue lines; dotted red lines indicate continuation) in the title compound.

## supplementary crystallographic information

### Comment

The Groebke–Blackburn reaction is the most popular way to prepare imidazo-azines from 2-aminoazines in a single-step (Groebke *et al.*, 1998; Bienaymé & Bouzid, 1998; Blackburn *et al.*, 1998). We have recently reported developments on this synthetic method (Nichol *et al.*, 2011; Sharma & Li, 2011) and present here the crystal structure of the title compound, determined as part of a larger study.

The asymmetric unit of the title compound is shown in Fig. 1. Molecular dimensions are unexceptional. Both ring systems are essentially planar (a mean plane fitted through atoms N1, N2, N3 C1 > C7 has an r.m.s. deviation of 0.019 Å; a mean plane fitted through atoms N3, C8 > C15 has an r.m.s. deviation of 0.016 Å) and the angle between both planes is 86.76 (2)°.

In the crystal, N—H···O interactions form a one-dimensional *C*^2^_2_(9) chain (Bernstein *et al.* 1995), which propagates in the *b*-axis direction (Fig. 2). C—H···O interactions are also found in the crystal packing.

### Experimental

The synthesis is described in Sharma & Li (2011).

### Refinement

All H atoms were located from a difference Fourier map and are freely refined. N—H distances are 0.869 (16) and 0.832 (16) Å; C—H distances lie in the range 0.911 (4)–1.033 (17) Å.

### Crystal data

**Table:**

C_15_H_16_N_3_^+^·ClO_4_^−^	*Z* = 2
*M**_r_* = 337.76	*F*(000) = 352
Triclinic, *P*1	*D*_x_ = 1.462 Mg m^−^^3^
Hall symbol: -P 1	Mo *K*α radiation, λ = 0.71073 Å
*a* = 8.6347 (3) Å	Cell parameters from 8544 reflections
*b* = 8.7663 (3) Å	θ = 2.5–39.0°
*c* = 11.5155 (4) Å	µ = 0.27 mm^−^^1^
α = 70.668 (2)°	*T* = 100 K
β = 73.131 (2)°	Block, colourless
γ = 72.679 (2)°	0.26 × 0.16 × 0.16 mm
*V* = 767.24 (5) Å^3^	

### Data collection

**Table:**

Bruker Kappa APEXII DUO CCD diffractometer	8640 independent reflections
Radiation source: fine-focus sealed tube with Miracol optics	6871 reflections with *I* > 2σ(*I*)
graphite	*R*_int_ = 0.031
φ and ω scans	θ_max_ = 38.6°, θ_min_ = 1.9°
Absorption correction: multi-scan (*SADABS*; Sheldrick, 1996)	*h* = −13→15
*T*_min_ = 0.932, *T*_max_ = 0.957	*k* = −15→15
27864 measured reflections	*l* = −20→20

### Refinement

**Table:**

Refinement on *F*^2^	Primary atom site location: structure-invariant direct methods
Least-squares matrix: full	Secondary atom site location: difference Fourier map
*R*[*F*^2^ > 2σ(*F*^2^)] = 0.038	Hydrogen site location: difference Fourier map
*wR*(*F*^2^) = 0.111	All H-atom parameters refined
*S* = 1.05	*w* = 1/[σ^2^(*F*_o_^2^) + (0.0581*P*)^2^ + 0.1118*P*] where *P* = (*F*_o_^2^ + 2*F*_c_^2^)/3
8640 reflections	(Δ/σ)_max_ = 0.001
272 parameters	Δρ_max_ = 0.61 e Å^−^^3^
0 restraints	Δρ_min_ = −0.51 e Å^−^^3^

### Special details

**Table:**

Geometry. All e.s.d.'s (except the e.s.d. in the dihedral angle between two l.s. planes) are estimated using the full covariance matrix. The cell e.s.d.'s are taken into account individually in the estimation of e.s.d.'s in distances, angles and torsion angles; correlations between e.s.d.'s in cell parameters are only used when they are defined by crystal symmetry. An approximate (isotropic) treatment of cell e.s.d.'s is used for estimating e.s.d.'s involving l.s. planes.
Refinement. Refinement of *F*^2^ against ALL reflections. The weighted *R*-factor *wR* and goodness of fit *S* are based on *F*^2^, conventional *R*-factors *R* are based on *F*, with *F* set to zero for negative *F*^2^. The threshold expression of *F*^2^ > σ(*F*^2^) is used only for calculating *R*-factors(gt) *etc*. and is not relevant to the choice of reflections for refinement. *R*-factors based on *F*^2^ are statistically about twice as large as those based on *F*, and *R*-factors based on ALL data will be even larger.

### Fractional atomic coordinates and isotropic or equivalent isotropic displacement parameters (Å^2^)

**Table:**

	*x*	*y*	*z*	*U*_iso_*/*U*_eq_	
N1	0.98956 (8)	0.60185 (8)	0.27803 (6)	0.01285 (10)	
N2	0.85450 (9)	0.82744 (9)	0.33586 (7)	0.01631 (12)	
H2N	0.825 (2)	0.904 (2)	0.3751 (15)	0.032 (4)*	
N3	0.82987 (9)	0.59792 (9)	0.13756 (7)	0.01541 (11)	
H3N	0.782 (2)	0.5193 (19)	0.1717 (14)	0.030 (4)*	
C1	1.10989 (10)	0.45913 (10)	0.27281 (8)	0.01559 (13)	
H1	1.1040 (18)	0.3970 (17)	0.2222 (13)	0.021 (3)*	
C2	1.22970 (11)	0.41819 (11)	0.33968 (8)	0.01768 (14)	
H2	1.3107 (17)	0.3243 (17)	0.3371 (13)	0.021 (3)*	
C3	1.23050 (11)	0.52108 (11)	0.41221 (8)	0.01805 (14)	
H3	1.3177 (18)	0.4918 (18)	0.4584 (14)	0.026 (3)*	
C4	1.11017 (11)	0.66267 (11)	0.41743 (8)	0.01665 (13)	
H4	1.1091 (17)	0.7333 (17)	0.4609 (13)	0.022 (3)*	
C5	0.98717 (10)	0.70098 (9)	0.34900 (7)	0.01399 (12)	
C6	0.77067 (10)	0.80959 (10)	0.25805 (8)	0.01618 (13)	
H6	0.6720 (18)	0.8831 (18)	0.2376 (14)	0.025 (3)*	
C7	0.85317 (10)	0.66985 (9)	0.22032 (7)	0.01359 (12)	
C8	0.77759 (10)	0.70835 (9)	0.02641 (7)	0.01420 (12)	
C9	0.62087 (11)	0.72027 (11)	0.00791 (8)	0.01716 (13)	
C10	0.57589 (12)	0.82519 (12)	−0.10399 (9)	0.02081 (15)	
H10	0.4688 (18)	0.8271 (17)	−0.1195 (13)	0.023 (3)*	
C11	0.68217 (13)	0.91810 (12)	−0.19433 (9)	0.02220 (16)	
H11	0.657 (2)	0.9887 (19)	−0.2776 (15)	0.033 (4)*	
C12	0.83699 (12)	0.90525 (11)	−0.17397 (8)	0.02083 (15)	
H12	0.915 (2)	0.9649 (19)	−0.2370 (15)	0.032 (4)*	
C13	0.88820 (11)	0.79974 (10)	−0.06457 (8)	0.01662 (13)	
C14	0.50193 (13)	0.62394 (14)	0.10725 (10)	0.02599 (19)	
H14A	0.549 (2)	0.498 (2)	0.1225 (15)	0.035 (4)*	
H14B	0.483 (2)	0.655 (2)	0.1867 (16)	0.037 (4)*	
H14C	0.394 (2)	0.652 (2)	0.0884 (16)	0.037 (4)*	
C15	1.05888 (12)	0.78164 (13)	−0.04629 (9)	0.02178 (16)	
H15A	1.0548 (18)	0.8453 (18)	0.0085 (14)	0.027 (4)*	
H15B	1.135 (2)	0.819 (2)	−0.1279 (16)	0.036 (4)*	
H15C	1.1063 (19)	0.6657 (19)	−0.0109 (14)	0.028 (4)*	
Cl	0.65990 (2)	0.20252 (2)	0.419315 (17)	0.01454 (5)	
O1	0.79691 (9)	0.07166 (9)	0.46174 (7)	0.02290 (13)	
O2	0.60990 (9)	0.15985 (9)	0.32753 (7)	0.02488 (14)	
O3	0.71409 (13)	0.35550 (10)	0.36274 (8)	0.03427 (19)	
O4	0.52640 (10)	0.21967 (12)	0.52460 (7)	0.03483 (19)	

### Atomic displacement parameters (Å^2^)

**Table:**

	*U*^11^	*U*^22^	*U*^33^	*U*^12^	*U*^13^	*U*^23^
N1	0.0136 (3)	0.0125 (2)	0.0123 (2)	−0.0019 (2)	−0.0038 (2)	−0.0031 (2)
N2	0.0182 (3)	0.0136 (3)	0.0171 (3)	−0.0008 (2)	−0.0050 (2)	−0.0054 (2)
N3	0.0204 (3)	0.0135 (3)	0.0136 (3)	−0.0049 (2)	−0.0072 (2)	−0.0011 (2)
C1	0.0161 (3)	0.0143 (3)	0.0155 (3)	−0.0001 (2)	−0.0046 (2)	−0.0047 (2)
C2	0.0157 (3)	0.0180 (3)	0.0183 (3)	0.0004 (3)	−0.0061 (3)	−0.0049 (3)
C3	0.0164 (3)	0.0210 (3)	0.0175 (3)	−0.0037 (3)	−0.0065 (3)	−0.0042 (3)
C4	0.0184 (3)	0.0180 (3)	0.0156 (3)	−0.0050 (3)	−0.0055 (3)	−0.0047 (3)
C5	0.0153 (3)	0.0135 (3)	0.0133 (3)	−0.0030 (2)	−0.0035 (2)	−0.0037 (2)
C6	0.0163 (3)	0.0144 (3)	0.0167 (3)	−0.0006 (2)	−0.0054 (2)	−0.0037 (2)
C7	0.0141 (3)	0.0132 (3)	0.0131 (3)	−0.0020 (2)	−0.0047 (2)	−0.0024 (2)
C8	0.0166 (3)	0.0133 (3)	0.0125 (3)	−0.0031 (2)	−0.0051 (2)	−0.0018 (2)
C9	0.0175 (3)	0.0185 (3)	0.0154 (3)	−0.0046 (3)	−0.0059 (3)	−0.0018 (3)
C10	0.0213 (4)	0.0218 (4)	0.0188 (3)	−0.0018 (3)	−0.0104 (3)	−0.0020 (3)
C11	0.0290 (4)	0.0189 (3)	0.0161 (3)	−0.0023 (3)	−0.0094 (3)	0.0000 (3)
C12	0.0270 (4)	0.0178 (3)	0.0149 (3)	−0.0072 (3)	−0.0039 (3)	0.0006 (3)
C13	0.0187 (3)	0.0157 (3)	0.0148 (3)	−0.0048 (3)	−0.0028 (2)	−0.0030 (2)
C14	0.0204 (4)	0.0336 (5)	0.0227 (4)	−0.0128 (4)	−0.0065 (3)	0.0016 (4)
C15	0.0186 (4)	0.0249 (4)	0.0222 (4)	−0.0087 (3)	−0.0026 (3)	−0.0048 (3)
Cl	0.01653 (8)	0.01267 (7)	0.01320 (7)	−0.00157 (5)	−0.00390 (6)	−0.00279 (5)
O1	0.0223 (3)	0.0221 (3)	0.0274 (3)	0.0056 (2)	−0.0147 (3)	−0.0118 (3)
O2	0.0258 (3)	0.0247 (3)	0.0311 (4)	0.0006 (3)	−0.0171 (3)	−0.0124 (3)
O3	0.0591 (6)	0.0231 (3)	0.0265 (4)	−0.0231 (4)	−0.0188 (4)	0.0067 (3)
O4	0.0263 (4)	0.0410 (5)	0.0207 (3)	0.0058 (3)	0.0040 (3)	−0.0066 (3)

### Geometric parameters (Å, °)

**Table:**

N1—C1	1.3741 (10)	C8—C13	1.4039 (11)
N1—C5	1.3687 (10)	C9—C10	1.3966 (12)
N1—C7	1.3992 (10)	C9—C14	1.5071 (13)
N2—H2N	0.869 (16)	C10—H10	0.985 (14)
N2—C5	1.3408 (11)	C10—C11	1.3849 (14)
N2—C6	1.3740 (11)	C11—H11	0.999 (16)
N3—H3N	0.832 (16)	C11—C12	1.3891 (14)
N3—C7	1.3896 (10)	C12—H12	0.966 (16)
N3—C8	1.4262 (10)	C12—C13	1.3951 (12)
C1—H1	0.939 (14)	C13—C15	1.5034 (13)
C1—C2	1.3602 (12)	C14—H14A	1.033 (17)
C2—H2	0.911 (14)	C14—H14B	0.994 (17)
C2—C3	1.4203 (12)	C14—H14C	0.958 (17)
C3—H3	0.971 (15)	C15—H15A	0.958 (15)
C3—C4	1.3671 (12)	C15—H15B	0.994 (16)
C4—H4	0.912 (14)	C15—H15C	0.971 (15)
C4—C5	1.3999 (11)	Cl—O1	1.4528 (7)
C6—H6	0.941 (15)	Cl—O2	1.4375 (7)
C6—C7	1.3601 (11)	Cl—O3	1.4353 (8)
C8—C9	1.3985 (11)	Cl—O4	1.4239 (8)
			
C1—N1—C5	121.77 (7)	C8—C9—C10	118.57 (8)
C1—N1—C7	129.50 (7)	C8—C9—C14	120.81 (7)
C5—N1—C7	108.71 (6)	C10—C9—C14	120.61 (8)
H2N—N2—C5	123.8 (11)	C9—C10—H10	118.3 (8)
H2N—N2—C6	126.6 (11)	C9—C10—C11	121.14 (8)
C5—N2—C6	109.48 (7)	H10—C10—C11	120.4 (8)
H3N—N3—C7	114.8 (11)	C10—C11—H11	123.1 (9)
H3N—N3—C8	114.5 (11)	C10—C11—C12	119.44 (8)
C7—N3—C8	116.63 (7)	H11—C11—C12	117.3 (9)
N1—C1—H1	117.3 (9)	C11—C12—H12	121.1 (9)
N1—C1—C2	118.21 (7)	C11—C12—C13	121.33 (8)
H1—C1—C2	124.5 (9)	H12—C12—C13	117.5 (9)
C1—C2—H2	119.5 (9)	C8—C13—C12	118.23 (8)
C1—C2—C3	120.71 (8)	C8—C13—C15	121.02 (7)
H2—C2—C3	119.8 (9)	C12—C13—C15	120.74 (8)
C2—C3—H3	120.1 (9)	C9—C14—H14A	111.5 (9)
C2—C3—C4	120.84 (7)	C9—C14—H14B	108.0 (10)
H3—C3—C4	119.1 (9)	C9—C14—H14C	113.1 (10)
C3—C4—H4	123.1 (9)	H14A—C14—H14B	109.3 (13)
C3—C4—C5	117.33 (8)	H14A—C14—H14C	109.5 (14)
H4—C4—C5	119.6 (9)	H14B—C14—H14C	105.1 (14)
N1—C5—N2	107.30 (7)	C13—C15—H15A	110.8 (9)
N1—C5—C4	121.11 (7)	C13—C15—H15B	111.2 (10)
N2—C5—C4	131.58 (8)	C13—C15—H15C	109.7 (9)
N2—C6—H6	123.8 (9)	H15A—C15—H15B	108.4 (13)
N2—C6—C7	108.34 (7)	H15A—C15—H15C	109.6 (12)
H6—C6—C7	127.8 (9)	H15B—C15—H15C	107.1 (13)
N1—C7—N3	120.77 (7)	O1—Cl—O2	108.58 (4)
N1—C7—C6	106.16 (7)	O1—Cl—O3	109.13 (5)
N3—C7—C6	132.97 (7)	O1—Cl—O4	109.21 (5)
N3—C8—C9	120.05 (7)	O2—Cl—O3	109.54 (5)
N3—C8—C13	118.66 (7)	O2—Cl—O4	110.96 (6)
C9—C8—C13	121.27 (7)	O3—Cl—O4	109.39 (6)
			
C5—N1—C1—C2	−0.63 (12)	C1—N1—C7—C6	178.53 (8)
C7—N1—C1—C2	−179.04 (8)	C5—N1—C7—N3	176.88 (7)
N1—C1—C2—C3	−0.38 (13)	C5—N1—C7—C6	−0.04 (9)
C1—C2—C3—C4	0.61 (13)	C7—N3—C8—C9	−114.31 (9)
C2—C3—C4—C5	0.16 (13)	C7—N3—C8—C13	67.62 (10)
C6—N2—C5—N1	0.59 (9)	N3—C8—C9—C10	−177.75 (8)
C6—N2—C5—C4	−179.96 (9)	N3—C8—C9—C14	3.10 (13)
C1—N1—C5—N2	−179.04 (7)	C13—C8—C9—C10	0.27 (13)
C1—N1—C5—C4	1.44 (12)	C13—C8—C9—C14	−178.88 (9)
C7—N1—C5—N2	−0.34 (9)	C8—C9—C10—C11	−1.10 (14)
C7—N1—C5—C4	−179.86 (7)	C14—C9—C10—C11	178.05 (10)
C3—C4—C5—N1	−1.16 (12)	C9—C10—C11—C12	0.78 (15)
C3—C4—C5—N2	179.45 (9)	C10—C11—C12—C13	0.40 (15)
C5—N2—C6—C7	−0.62 (10)	C11—C12—C13—C8	−1.19 (14)
N2—C6—C7—N1	0.39 (9)	C11—C12—C13—C15	177.38 (9)
N2—C6—C7—N3	−175.99 (8)	N3—C8—C13—C12	178.90 (8)
C8—N3—C7—N1	−138.11 (8)	N3—C8—C13—C15	0.33 (12)
C8—N3—C7—C6	37.84 (13)	C9—C8—C13—C12	0.85 (13)
C1—N1—C7—N3	−4.55 (12)	C9—C8—C13—C15	−177.71 (8)

### Hydrogen-bond geometry (Å, °)

**Table:**

*D*—H···*A*	*D*—H	H···*A*	*D*···*A*	*D*—H···*A*
N2—H2N···O1^i^	0.869 (16)	1.955 (17)	2.8169 (10)	170.8 (15)
N3—H3N···O3	0.832 (16)	2.216 (15)	2.8899 (10)	138.3 (14)
C2—H2···O2^ii^	0.911 (14)	2.547 (14)	3.3826 (11)	152.8 (12)
C3—H3···O4^ii^	0.971 (15)	2.559 (15)	3.2893 (12)	132.0 (11)

Symmetry codes: (i) *x*, *y*+1, *z*; (ii) *x*+1, *y*, *z*.
